# Postinspiratory and preBötzinger complexes contribute to respiratory-sympathetic coupling in mice before and after chronic intermittent hypoxia

**DOI:** 10.3389/fnins.2024.1386737

**Published:** 2024-05-06

**Authors:** Marlusa Karlen-Amarante, Zachary T. Glovak, Alyssa Huff, Luiz M. Oliveira, Jan-Marino Ramirez

**Affiliations:** ^1^Center for Integrative Brain Research, Seattle Children’s Research Institute, Seattle, WA, United States; ^2^Department of Neurological Surgery, University of Washington, Seattle, WA, United States

**Keywords:** chronic intermittent hypoxia, sympathetic nerve activity, post-inspiratory complex, preBötzinger complex, optogenetics

## Abstract

The sympathetic nervous system modulates arterial blood pressure. Individuals with obstructive sleep apnea (OSA) experience numerous nightly hypoxic episodes and exhibit elevated sympathetic activity to the cardiovascular system leading to hypertension. This suggests that OSA disrupts normal respiratory-sympathetic coupling. This study investigates the role of the postinspiratory complex (PiCo) and preBötzinger complex (preBötC) in respiratory-sympathetic coupling under control conditions and following exposure to chronic intermittent hypoxia (CIH) for 21 days (5% O_2_–80 bouts/day). The surface of the ventral brainstem was exposed in urethane (1.5 g/kg) anesthetized, spontaneously breathing adult mice. Cholinergic (ChAT), glutamatergic (Vglut2), and neurons that co-express ChAT and Vglut2 at PiCo, as well as Dbx1 and Vglut2 neurons at preBötC, were optogenetically stimulated while recording activity from the diaphragm (DIA), vagus nerve (cVN), and cervical sympathetic nerve (cSN). Following CIH exposure, baseline cSN activity increased, breathing frequency increased, and expiratory time decreased. In control mice, stimulating PiCo specific cholinergic-glutamatergic neurons caused a sympathetic burst during all phases of the respiratory cycle, whereas optogenetic activation of cholinergic-glutamatergic PiCo neurons in CIH mice increased sympathetic activity only during postinspiration and late expiration. Stimulation of glutamatergic PiCo neurons increased cSN activity during the postinspiratory phase in control and CIH mice. Optogenetic stimulation of ChAT containing neurons in the PiCo area did not affect sympathetic activity under control or CIH conditions. Stimulating Dbx1 or Vglut2 neurons in preBötC evoked an inspiration and a concomitant cSN burst under control and CIH conditions. Taken together, these results suggest that PiCo and preBötC contribute to respiratory-sympathetic coupling, which is altered by CIH, and may contribute to the hypertension observed in patients with OSA.

## 1 Introduction

Obstructive sleep apnea (OSA) represents a significant public health burden and is increasing in prevalence (Franklin and Lindberg, 2015). Cardiopulmonary sequelae of OSA include arterial hypertension, increased ventilatory response to hypercapnia, endothelial dysfunction, atherosclerosis, and disordered swallow-breathing coordination (Fletcher, 2001; Prabhakar et al., 2005; Schulz et al., 2005; Nanduri et al., 2012; Dumitrascu et al., 2013; Huff et al., 2022). Chronic intermittent hypoxia (CIH) is used as an experimental paradigm to model the pathophysiological changes associated with OSA. In rats, CIH increases sympathetic activity and disrupts respiratory-sympathetic coupling resulting in hypertension (Zoccal et al., 2008; Simms et al., 2009; Zoccal et al., 2009; Molkov et al., 2011). Furthermore, when exposure to CIH occurs postnatally, elevated arterial pressure levels and baroreflex dysfunctions can persist into adulthood (Soukhova-O'Hare et al., 2006; Karlen-Amarante et al., 2023). The cardiopulmonary effects of CIH have been attributed to the overexcitation of rostral ventrolateral medulla (RVLM) presympathetic neurons (Karlen-Amarante et al., 2023) via excitatory and inhibitory inputs from respiratory rhythmogenic networks (Lipski et al., 1996; Molkov et al., 2011; Moraes et al., 2012, 2013). However, the exact neuronal mechanisms underlying respiratory modulation of sympathetic activity remain to be characterized.

To investigate the role of central respiratory networks in sympathetic control and how their function is altered by CIH in mice, we focused on two respiratory networks located within the medulla: the postinspiratory complex (PiCo) and the pre-Bötzinger complex (preBötC). The preBötC generates inspiratory activity (Anderson and Ramirez, 2017), and contains excitatory, glutamatergic inspiratory neurons (vesicular glutamate transporter 2; Vglut2) that are primarily derived from progenitor cells characterized by the transcription factor Dbx1 (Bouvier et al., 2010; Gray et al., 2010; Cui et al., 2016). It is well-known that preBötC also regulates cardiorespiratory oscillations in sympathetic activity by providing excitatory input to presympathetic catecholaminergic neurons in the RVLM (Moraes et al., 2013; Menuet et al., 2017, 2020). In contrast, the extent to which PiCo contributes to respiratory-sympathetic coupling in mice has not been investigated. PiCo contains neurons that co-express markers for cholinergic (ChAT) and glutamatergic (Vglut2) neurotransmitters (Anderson et al., 2016) and is important for the control of postinspiratory activity (Anderson et al., 2016) and swallow-breathing coordination (Huff et al., 2023). Chemical inhibition of PiCo has been shown to reduce sympathetic output in rats (Toor et al., 2019). Thus, the present study tested the hypothesis that PiCo and preBötC modulate respiratory-sympathetic coupling in mice, which is altered by CIH.

## 2 Methods

### 2.1 Ethical approval and transgenic mice

All experiments and procedures were approved by the Seattle Children’s Research Institute (SCRI) Institutional Animal Care and Use Committee (IACUC) and conducted in accordance with the National Institutes of Health and ARRIVE guidelines (Percie du Sert et al., 2020). Adult (P42 to P152), female (*n* = 32) and male (*n* = 34) mice used in this study were bred at SCRI and housed in a temperature (22 ± 1°C) and humidity (25–75%) controlled room with a 12:12 h light–dark cycle. Mice were provided with a standard diet and water *ad libitum*.

Although many studies of the cardiopulmonary effects of CIH have utilized rats, the present study leverages the genetic homology mice have with humans (O'Brien et al., 1999) to advance understanding of the pathogenesis of OSA (Nanduri et al., 2012; Barnes et al., 2022). All the following Cre mice were crossed with homozygous mice containing a floxed STOP channelrhodopsin-2 (ChR2) fused to a EYFP (Ai32) reporter sequence from Jackson Laboratories (stock number 024109). Vglut2-IRES-cre (Vglut2), Vgat-IRES-cre (Vgat), and ChAT-IRES-cre (ChAT) homozygous breeder lines were obtained from Jackson Laboratories (stock numbers 028863; 016962, and 031661 respectively). Heterozygous Dbx1creERT2 mice were donated by Dr. Christopher Del Negro (College of William and Mary, VA) and a homozygous breeder line was generated at SCRI. Homozygous Dbx1creERT2 dams were plug checked and injected intraperitoneally (IP) at E10.5 with tamoxifen (24 mg/kg) to target preBötC Dbx1 neurons (Baertsch et al., 2019; Huff et al., 2022). ChAT-IRES-cre(Δneo), technically known as 129S-Chat^tm1(cre)Lowl^/MwarJ strain, and Vglut2-IRES2-FlpO-D, technically known as 129S-Slc17a6^tm1.1(flpo)Hze^/J were obtained from Jackson Laboratories (stock numbers 031661 and 030212, respectively). To generate double-transgenic mice, the ChATcre and Vglut2FlpO strains were interbred to generate compound homozygotes, referred to as ChATcre:Vglut2FlpO, in which neurons that have a developmental history of expressing both cholinergic and glutamatergic neurotransmitters express both Cre and Flp recombinases. Experimental groups are referred to as Vglut2cre:Ai32, Vgatcre:Ai32, ChATcre:Ai32, Dbx1cre:Ai32, and ChATcre:Vglut2FlpO:ChR2. All mice used in this study were on the C57BL/6 J (B6) background or back crossed with B6 mice (Vgatcre:Ai32 and Dbx1cre:Ai32).

### 2.2 Brainstem injection of adeno-associated virus (AAV)

All surgical procedures were performed under aseptic conditions. For targeted adeno-associated virus (AAV) injection, the mice (P42–131) were anesthetized with isoflurane (2%). Adequate anesthesia was confirmed by the absence of hind-paw withdrawal reflex. The hair over the skull was removed and the skin was disinfected. The mice were then placed prone on a stereotaxic apparatus (bite bar set at −3.5 mm; David Kopf Instruments, Tujunga, CA, USA). A bilateral 0.5 mm diameter craniotomy was drilled into the occipital bone caudal to lambda. Viral solutions were loaded into a 1.2 mm internal diameter glass pipette with a 20 μm tip (external diameter). To target the PiCo region with ChR2, the pipette was inserted into the brainstem at the following coordinates: 1.6 mm caudal to lambda, 1.1 mm lateral to the midline, and 4.8 mm below the dorsal surface of the cerebellum. Bilateral 150 nL injections of pAAV-hSyn Con/Fon hChR2(H134R)-EYFP adenovirus vector (cat# 55645-AAV8; AddGene, USA; abbreviated as AAV8-ConFon-ChR2-EYFP) were made at a rate of 50 nL/min using a glass micropipette and an automatic nanoliter injector (Nanoject II, Drummond Scientific Co., Broomall, PA). This AAV is a Cre-on/Flp-on ChR2-EYFP under the synapsin promoter and encodes the photoactivatable cation channel channelrhodopsin-2 (ChR2, H134R) fused to EYFP. The vector was diluted to a final titer of 1 × 10^13^ viral particles/ml with sterile phosphate-buffered saline. Afterward, the skin was sutured, and the animals were treated with ketoprofen (5 mg/kg/day for 2 days). No abnormal behaviors were observed during the recovery period. Mice were given three days to recover from intracranial viral injections before being randomly assigned to CIH or normoxia protocols.

### 2.3 Chronic intermittent hypoxia (CIH) exposure

Offspring from optogenetic lines were randomly assigned to CIH or normoxia (control) protocols. The CIH protocol used in the present study has been previously described (Huff et al., 2022). Briefly, mouse cages were kept inside a custom-built dual oxygen/nitrogen cycling chamber (Oxycycler, Huff Technologies Inc., Morganfield, KY). One chamber was used for the control group and another for the CIH group. Mice in the CIH chamber were exposed to intermittent episodes of hypoxia consisting of 1 min of nitrogen (N2) injection to reduce the percentage of inspired oxygen (O_2_) inside the chamber from 21% to 4.5–5%. Then, compressed room air was injected into the chamber to return the percentage of O_2_ to 21% for 5 min before starting a new cycle of hypoxia. The entire 6 min CIH protocol was repeated 80 times per day over 8 h during the mouse’s subjective night. For the remaining 16 h, CIH mice were kept under normoxic conditions (21% O_2_). The injection of N2 and compressed air into the chambers was automated by custom computer software (Huff Technologies Inc.) and monitored by O_2_ sensors inside the chambers. The gas injection occurred at the top of the chamber to avoid potential stress on the mice. Directly below the CIH chamber, control mice were kept in an identical chamber under normoxic conditions 24 h a day for 21 days. All mice had *ad libitum* access to food and water during both protocols.

### 2.4 *In-vivo* preparation

After CIH or normoxia exposure, mice from both experimental groups were anesthetized with urethane (1.5 g/kg, IP) and placed supine on a custom surgical table with a heating pad to keep mouse body temperature constant at 37°C. Adequate depth of anesthesia was confirmed every 15 min via heart and breathing rates and absence of hind-paw withdrawal reflex. The trachea was exposed through a midline incision and cannulated caudal to the larynx with a curved (180 degree) tracheal tube (24 G). A line delivering 100% O_2_ was attached to the cannulated trachea to provide O_2_ throughout the experiment. All the recorded nerves were isolated unilaterally, cut distally, and recorded via a unipolar suction electrode connected to a fire-polished, pulled glass pipette filled with aCSF. The corresponding contralateral nerve was preserved and remained intact. The cervical sympathetic nerve (cSN) was isolated and cut caudal to the superior cervical sympathetic ganglion. The cervical vagus (cVN) nerve was isolated, and the trachea and esophagus were cut and detached at the rostral end. The occipital bone was removed, and the ventral medullary surface was bathed with warm (~36°C) artificial cerebrospinal fluid (aCSF; in mM: 118 NaCl, 3 KCl, 25 NaHCO_3_, 1 NaH_2_PO_4_, 1 MgCl_2_, 1.5 CaCl_2_, 30 D-glucose) equilibrated with carbogen (95% O_2_, 5% CO_2_) by a peristaltic pump (Dynamax RP-1, Rainin Instrument Co, Emeryville CA, USA). A bipolar electromyogram (EMG) electrode (A-M systems, Carlsborg, USA) was placed in the costal diaphragm muscle (DIA) to record the inspiratory activity and monitor anesthetic depth. The cSN and cVN nerve efferences were recorded using glass suction unipolar electrodes secured in a 3D micromanipulator (Narishige, Tokyo, Japan). All EMG and electroneurogram (ENG) signals were acquired with a A/D converter, (CED micro 1401, Cambridge Electronic Design, Cambridge, UK) amplified (AC Amplifier, model 1700, A-M System, Carlsborg, USA), and band-pass filtered (100–3 kHz). Using Spike 2 software (5 KHz, CED, Cambridge, UK), data was further processed using a band pass filter (200–700 Hz, 40 Hz transition gap), rectified, and smoothed (20 ms).

### 2.5 Optogenetic stimulation and data analysis

Optical fibers with a diameter of 200 μm were connected to a blue (470 nm) high-powered LED (ThorLabs Inc., Newton, NJ, USA) and positioned bilaterally on the ventral surface above preBötC or PiCo by utilizing the vertebral and basilar arteries as landmarks as described in previous studies (Anderson et al., 2016; Baertsch et al., 2018; Huff et al., 2022, 2023). Power was set at 0.75 mW/mm^2^. To determine the probability of optogenetically evoking diaphragm bursts and sympathetic activity responses, 200 ms light pulses were triggered every 10 s for 4 min (total 25 stims) to stimulate ChAT, Vglut2, and neurons that co-express ChAT and Vglut2 in PiCo or Dbx1 and Vglut2 neurons in preBötC. This resulted in the stimulations occurring randomly across the respiratory cycle. To avoid artificially inflating degrees of freedom, stimulations were averaged within each respiratory phase within each mouse. In Vgat:Ai32 mice, continuous 10 s light pulses were triggered every 60 s to stimulate GABAergic neurons in preBötC.

The frequency of DIA discharge, or respiratory rate (RR), was determined by measuring the time interval over 10 consecutive bursts (expressed as bursts per minute). We quantified the DIA burst duration (time of inspiration, s) and interval between inspiratory discharges (time of expiration, s). Heart rate (HR) was derived from the DIA EMG. Spike 2 was used to detect and analyze the number of spikes with a half-width of 0.5 ms present in the cSN raw signal. In addition, the distribution of sympathetic discharge throughout the respiratory cycle was calculated to quantify the phase-specificity of respiratory-sympathetic coupling. The inspiratory phase was determined by the duration of the DIA burst. Expiration was divided into two phases based on the vagus activity: postinspiration and late expiration.

The cSN activity was recorded and analyzed in absolute units (volts, V). Noise levels were quantified at the end of each experiment and subtracted from the cSN activity. The integrated sympathetic activity signal was used to calculate the amplitude of the responses to the optogenetic stimulation. The amplitude of the cSN recordings were normalized to allow for comparisons between mice. During each mouse’s respective baseline, the peak cSN amplitudes during 10 respiratory cycles were standardized to the highest peak of normal physiological cSN discharge during the respiratory cycles and expressed as a percentage of the maximum cSN peak (% MAX). Then, optogenetically evoked responses in cSN activity were expressed as % MAX of baseline cSN maximum amplitude during inspiration. We have previously shown that stimulation of PiCo neurons has a high probability of evoking a swallow in a respiratory-phase dependent manner (Huff et al., 2023). To evaluate the PiCo contribution to respiratory-sympathetic coupling, only PiCo stimulations that did not trigger a swallow were included in this study.

### 2.6 Histology

At the end of the experiments, all animals were deeply anesthetized with 5% isoflurane in 100% oxygen and perfused through the ascending aorta with 20 mL of phosphate buffered saline (PB; pH 7.4) followed by 4% phosphate-buffered (0.1 M; pH 7.4; 20 mL) paraformaldehyde (Electron Microscopy Sciences, Fort Washington, PA). The brains were removed and stored in the perfusion fixative for 4 h at 4°C followed by 20% sucrose for 8 h. Coronal brain sections (25 μm) were serially cut using a cryostat and stored in cryoprotectant solution at −20°C (20% glycerol plus 30% ethylene glycol in 50 mL phosphate buffer, pH 7.4) prior to histological processing. All histochemical procedures were done using free-floating sections.

ChAT was detected using a polyclonal goat anti-ChAT antibody (AB144P; Millipore; 1:100) and EYFP was detected using a polyclonal mouse anti-GFP (06–896, Millipore; 1:1000) diluted in PB containing 2% normal donkey serum (017-000-121, Jackson Immuno Research Laboratories) and 0.3% Triton X-100, incubated for 24 h. Sections were subsequently rinsed in PB and incubated for 2 h in an Alexa 488 donkey anti-mouse antibody (715-545-150; 1:250; Jackson Immuno Research Laboratories) and Alexa 594 donkey anti-goat antibody (705-585-003; 1:250; Jackson Immuno Research Laboratories). For all secondary antibodies, control experiments confirmed that no labeling was observed when primary antibodies were omitted. The sections were mounted on slides in rostrocaudal sequential order, dried, and covered with fluoromount (00-4,958-02; Thermo Fisher). Coverslips were affixed with nail polish.

### 2.7 Cell counting, imaging, and data analysis

A VS120-S6-W Virtual Slide Scanner (Olympus; Tokyo, Japan) was used to scan all the sections. Images were taken with a color camera (Nikon DS-Fi3; Tokyo, Japan). To reduce bias, the photomicrography and counting were performed by a blinded researcher. Image J (version 1.41; National Institutes of Health, Bethesda, MD) was used for cell counting and Canvas software (ACD Systems, Victoria, Canada, v. 9.0) was used for line drawings. A one-in-two series of 25-μm brain sections was used per mouse, such that each section analyzed was 50 μm apart. The area analyzed was delimited (mean of 5,423 μm^2^) based on previous data (Anderson et al., 2016). Sections were examined to confirm the number of transfected cells. The number of labeled cells were bilaterally counted and reported as median ± interquartile range (IQR). Section alignment was relative to a reference section, as previously described (Anderson et al., 2016) and based on the Paxinos and Franklin atlas (Paxinos and Franklin, 2012).

### 2.8 Statistical analysis

Statistical analyses were performed using GraphPad Prism 8 (GraphPad, San Diego, CA). All data passed the Shapiro–Wilk test for normality. Two-way analysis of variance (ANOVA) followed by Bonferroni *post hoc* tests were used to test for phase-relationship responses. Unpaired, two-tailed t-tests were used to compare the baseline activity between control and CIH groups. The comparisons between baseline and optogenetic stimulation within the same group were made via repeated measures, two-way ANOVA or a mixed effects analysis (missing data). Differences were considered significant at *p* < 0.05. Investigators were not blinded during the analyses. Sample sizes were chosen based on previous studies (Anderson et al., 2016; Huff et al., 2022, 2023). Raw data summaries are presented in Tables 1–5. ANOVA tables are presented in Supplementary Table 1.

**Table 1:** Phase-dependent sympathetic parameters during baseline conditions and during optogenetic stimulation of cholinergic-glutamatergic PiCo neurons in control and CIH mice.

	Control (*n* = 5)					
	Stim I		Stim post-I		Stim E2	
	Baseline	Stim	Baseline	Stim	Baseline	Stim
A.U.C. (a.u.)	0.008 ± 0.005	0.011 ± 0.005	0.008 ± 0.002	0.009 ± 0.003	0.003 ± 0.002	0.011 ± 0.005^*^
Peak amplitude (V)	0.039 ± 0.008	0.069 ± 0.017^*^	0.017 ± 0.007	0.061 ± 0.022^*^	0.145 ± 0.082	0.057 ± 0.016^*^
Response duration (s)	0.19 ± 0.03	0.17 ± 0.03	0.462 ± 0.212	0.168 ± 0.030^*^	0.169 ± 0.056	0.114 ± 0.025
Amplitude % MAX (%)	77.60 ± 8.18	146.80 ± 53.79^*^	34.79 ± 15.61	131.0 ± 68.85^*^	29.01 ± 17.98	121.7 ± 51.94^*^
Heart rate (bpm)	413 ± 66	425 ± 68	412 ± 66	413 ± 68	413 ± 66	418 ± 73

	CIH (*n* = 5)					
	Stim I		Stim post-I		Stim E2	
	Baseline	Stim	Baseline	Stim	Baseline	Stim
A.U.C. (a.u.)	0.024 ± 0.022	0.026 ± 0.021	0.039 ± 0.064	0.049 ± 0.061	0.003 ± 0.001	0.007 ± 0.003^#^
Peak amplitude (V)	0.110 ± 0.070	0.165 ± 0.109	0.052 ± 0.038	0.131 ± 0.104^#^	0.023 ± 0.004	0.092 ± 0.035^#^
Response duration (s)	0.26 ± 0.11	0.21 ± 0.06	0.50 ± 0.32	0.22 ± 0.06	0.106 ± 0.038	0.127 ± 0.043
Amplitude % MAX (%)	81.36 ± 3.74	122.3 ± 18.71	39.89 ± 6.17	125.3 ± 61.20^#^	26.46 ± 7.91	140.10 ± 77.10^#^
Heart rate (bpm)	442 ± 56	457 ± 62	442 ± 56	443 ± 54	457 ± 58	457 ± 59

Values are mean ± SD. Data were analyzed via repeated measures, two-way ANOVA (one per row) and a Bonferroni post hoc test. Statistically significant differences caused by optogenetic stimulation are represented as follows: ^*^p < 0.05 from control baseline and ^#^*p* < 0.05 from CIH baseline. CON (*n* = 5, 2 male, 3 female); CIH (*n* = 5, 2 male, 3 female).

**Table 2:** Phase-dependent sympathetic parameters during baseline conditions and during optogenetic stimulation of glutamatergic PiCo neurons in control and CIH mice.

	Control (*n* = 6)					
	Stim I		Stim post-I		Stim E2	
	Baseline	Stim	Baseline	Stim	Baseline	Stim
A.U.C. (a.u.)	0.014 ± 0.008	0.012 ± 0.006	0.015 ± 0.006	0.013 ± 0.008	0.006 ± 0.002	0.005 ± 0.001
Peak amplitude (V)	0.102 ± 0.049	0.098 ± 0.042	0.037 ± 0.005	0.159 ± 0.126^*^	0.030 ± 0.007	0.058 ± 0.010
Response duration (s)	0.191 ± 0.063	0.154 ± 0.029	0.459 ± 0.219	0.121 ± 0.024^*^	0.181 ± 0.071	0.098 ± 0.018
Amplitude % MAX (%)	78.31 ± 8.50	78.49 ± 7.09	32.38 ± 10.95	128.50 ± 90.63^*^	27.08 ± 12.82	51.62 ± 16.93
Heart rate (bpm)	440 ± 65	445 ± 68	440 ± 65	446 ± 69	440 ± 65	445 ± 67

	CIH (*n* = 6)					
	Stim I		Stim post-I		Stim E2	
	Baseline	Stim	Baseline	Stim	Baseline	Stim
A.U.C. (a.u.)	0.012 ± 0.008	0.036 ± 0.047	0.011 ± 0.006	0.013 ± 0.009	0.003 ± 0.002	0.013 ± 0.015
Peak amplitude (V)	0.081 ± 0.058	0.093 ± 0.052	0.035 ± 0.024	0.129 ± 0.079^#^	0.027 ± 0.022	0.049 ± 0.037
Response duration (s)	0.231 ± 0.074	0.145 ± 0.043^#^	0.402 ± 0.153	0.142 ± 0.049^#^	0.123 ± 0.048	0.113 ± 0.060
Amplitude % MAX (%)	81.40 ± 6.32	104.8 ± 36.58	37.12 ± 12.91	160.1 ± 143.1^#^	28.42 ± 11.49	70.47 ± 38.61
Heart rate (bpm)	411 ± 92	415 ± 81	411 ± 92	413 ± 85	411 ± 92	412 ± 84

Values are mean ± SD. Data were analyzed via repeated measures, two-way ANOVA (one per row) and a Bonferroni post hoc test. Statistically significant differences caused by optogenetic stimulation are represented as follows: ^*^p < 0.05 from control baseline and ^#^*p* < 0.05 from CIH baseline. CON (*n* = 6, 5 male, 1 female); CIH (*n* = 6, 1 male, 5 female).

**Table 3:** Phase-dependent sympathetic parameters during baseline conditions and during optogenetic stimulation of cholinergic PiCo neurons in control and CIH mice.

	Control (*n* = 6)					
	Stim I		Stim post-I		Stim E2	
	Baseline	Stim	Baseline	Stim	Baseline	Stim
A.U.C. (a.u.)	0.014 ± 0.007	0.114 ± 0.007	0.021 ± 0.011	0.014 ± 0.009	0.008 ± 0.003	0.010 ± 0.002
Peak amplitude (V)	0.077 ± 0.029	0.009 ± 0.047	0.092 ± 0.084	0.146 ± 0.157	0.058 ± 0.048	0.090 ± 0.052
Response duration (s)	0.228 ± 0.063	0.155 ± 0.041	0.717 ± 0.281	0.179 ± 0.018^*^	0.262 ± 0.080	0.169 ± 0.024
Amplitude % MAX (%)	79.80 ± 14.46	104.50 ± 72.57	40.56 ± 20.39	110.3 ± 118.8	35.26 ± 22.34	93.12 ± 120.40
Heart rate (bpm)	367 ± 87	376 ± 87	375 ± 92	375 ± 92	379 ± 83	385 ± 86

	CIH (*n* = 5)					
	Stim I		Stim post-I		Stim E2	
	Baseline	Stim	Baseline	Stim	Baseline	Stim
A.U.C. (a.u.)	0.007 ± 0.004	0.017 ± 0.015	0.007 ± 0.004	0.014 ± 0.010	0.002 ± 0.001	0.008 ± 0.007
Peak amplitude (V)	0.054 ± 0.034	0.136 ± 0.113	0.032 ± 0.022	0.118 ± 0.110	0.025 ± 0.018	0.123 ± 0.113
Response duration (s)	0.188 ± 0.025	0.174 ± 0.039	0.303 ± 0.053	0.189 ± 0.021^#^	0.098 ± 0.017	0.090 ± 0.036
Amplitude % MAX (%)	81.42 ± 6.10	230.4 ± 157.4	47.82 ± 8.77	209.2 ± 178.9	39.65 ± 15.48	223.2 ± 208.8
Heart rate (bpm)	444 ± 70	444 ± 67	444 ± 70	441 ± 66	444 ± 70	442 ± 67

Values are mean ± SD. Data were analyzed via repeated measures, two-way ANOVA (one per row) and a Bonferroni post hoc test. Statistically significant differences caused by optogenetic stimulation are represented as follows: ^*^p < 0.05 from control baseline and ^#^*p* < 0.05 from CIH baseline. CON (*n* = 6, 4 male, 2 female); CIH (*n* = 5, 5 male).

**Table 4:** Sympathetic parameters during baseline conditions and during optogenetic stimulation of Dbx1 preBötC neurons in control and CIH mice.

	Control (*n* = 7)		CIH (*n* = 6)	
	Baseline	Stim	Baseline	Stim
A.U.C. (a.u.)	0.015 ± 0.006	0.027 ± 0.012^*^	0.022 ± 0.009	0.039 ± 0.013^#^
Peak amplitude (V)	0.062 ± 0.02	0.125 ± 0.06^*^	0.093 ± 0.04	0.178 ± 0.09^#^
Response duration (s)	0.22 ± 0.04	0.25 ± 0.10	0.20 ± 0.08	0.20 ± 0.05
Amplitude % MAX (%)	86 ± 3	137 ± 19^*^	83 ± 7	191 ± 44^#^
Heart rate (bpm)	402 ± 43	403 ± 42	459 ± 99	472 ± 110

Values are mean ± SD. Data were analyzed by paired, two-tailed t-tests. Statistically significant differences caused by optogenetic stimulation are represented as follows: ^*^p < 0.05 from control baseline and ^#^*p* < 0.05 from CIH baseline. CON (*n* = 7, 6 male, 1 female); CIH (*n* = 6, 1 male, 5 female).

**Table 5:** Sympathetic parameters during baseline conditions and optogenetic stimulation of glutamatergic preBötC neurons in control and CIH mice.

	Control (*n* = 8)		CIH (*n* = 7)	
	Baseline	Stim	Baseline	Stim
A.U.C. (a.u.)	0.028 ± 0.024	0.045 ± 0.024^*^	0.017 ± 0.013	0.040 ± 0.023^#^
Peak amplitude (V)	0.15 ± 0.13	0.26 ± 0.14^*^	0.092 ± 0.08	0.215 ± 0.13^#^
Response duration (s)	0.19 ± 0.05	0.19 ± 0.03	0.19 ± 0.04	0.21 ± 0.05
Amplitude % MAX (%)	85 ± 4	188 ± 66^*^	81 ± 11	269 ± 71^#^
Heart rate (bpm)	440 ± 57	438 ± 62	401 ± 124	423 ± 119

Values are mean ± SD. Data were analyzed by paired, two-tailed t-tests. Statistically significant differences caused by optogenetic stimulation are represented as follows: ^*^p < 0.05 from control baseline and ^#^*p* < 0.05 from CIH baseline. CON (*n* = 8, 5 male, 3 female); CIH (*n* = 7, 3 male, 4 female).

## 3 Results

### 3.1 Stimulating cholinergic-glutamatergic neurons at PiCo increases sympathetic activity in a respiratory phase-dependent manner

**Figure 1:**
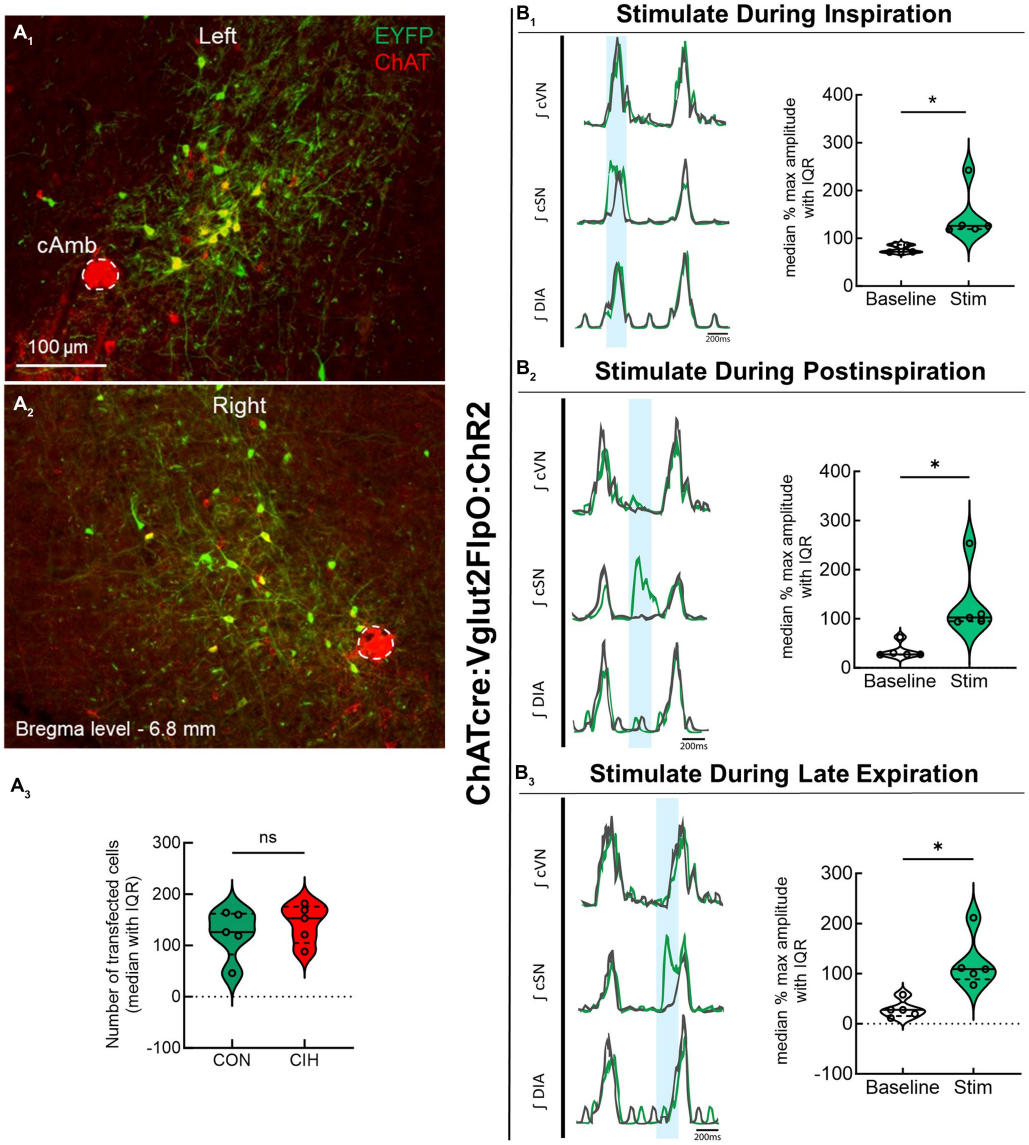
Stimulating cholinergic-glutamatergic neurons at PiCo increases sympathetic activity *in-vivo*. Representative transverse hemisections in a ChATcre:Vglut2FlpO:ChR2 mouse at −6.8 mm posterior to bregma illustrates selective bilateral transfection of cholinergic-glutamatergic neurons of PiCo left **(A**_**1**_**)** and right **(A**_**2**_**)** via the pAAv-hSyn Con/Fon hChR2 (H134R)-EYFP vector. Violin plots denote median (solid line) and interquartile range (IQR; dashed lines) and individual mouse data is illustrated via dots. Data distributions are denoted by the edges of the violins. Unpaired, two-tailed t-test indicated number of transfected cells was not different between control (CON) and chronic intermittent hypoxia (CIH) groups. Representative traces of raw and integrated (∫) diaphragm (DIA), cervical sympathetic nerve (cSN), and vagus nerve (cVN) are shown for a control ChATcre:Vglut2FlpO:ChR2 mouse during inspiration (1), postinspiration (2), and late expiration (3). The vertical shaded blue area in the trace shows the 200 ms LED activation, and the green trace illustrates the response to the optogenetic stimulation relative to the gray baseline trace within the same representative mouse. Repeated measures, two-way ANOVA with a Bonferroni *post hoc* test revealed optogenetic stimulation of cholinergic-glutamatergic neurons at PiCo caused significant increases in sympathetic nerve activity during inspiration **(B**_**1**_**)**, postinspiration **(B**_**2**_**)**, and late expiration phases **(B**_**3**_**)**. *p < 0.05; *n* = 5 (2 male, 3 female).

Previous studies have established that PiCo contains neurons that co-express the genetic markers for ChAT and Vglut2 (Anderson et al., 2016; Huff et al., 2022, 2023). To evaluate the role of PiCo specific neurons in respiratory-sympathetic coupling, neurons co-expressing ChAT and Vglut2 were targeted for optogenetic stimulation. Figure 1 shows neurons from transfected cells located just dorsal to the nucleus ambiguus near −6.84 mm posterior to Bregma, ~1.1 mm from the midline, and ~ 0.7 mm above the marginal layer according to the Paxinos and Franklin mouse atlas (2012). Neurons were targeted using an intersectional genetic strategy via bilateral injections of the vector AAV8-ConFon-ChR2-EYFP into PiCo region of ChATcre:Vglut2FlpO mice (Figure 1A_1_) left, (Figure 1A_2_) right. These cells were all ChAT-immunoreactive (ChAT-ir, red) and EYFP positive (green), indicating successful expression of channelrhodopsin. Nucleus ambiguus cholinergic neurons were not transfected (red, Figures 1A_1,2_). The median number of transfected neurons in both hemispheres was not significantly different between the control and CIH groups (Figure 1A_3_). Repeated measures two-way ANOVA revealed that *in-vivo* optogenetic stimulation (blue vertical bar) of ChAT-Vglut2 PiCo neurons produced significant (*F*(1, 4) = 9.981; *p* = 0.0342) increases in cSN activity (green trace vs. gray baseline trace) in a respiratory phase-dependent manner (*F*(2, 8) = 34.06; *p* < 0.0001). Bonferroni *post hoc* multiple comparisons test confirmed that optogenetic stimulation evoked a significant increase in sympathetic activity during inspiration (*p* < 0.0001; Figure 1B_1_), postinspiration (*p* < 0.0001; Figure 1B_2_), and late expiration (*p* < 0.0001; Figure 1B_3_). Heart rate was unaffected (Table 1).

### 3.2 Optogenetic stimulation of glutamatergic, but not cholinergic, neurons at PiCo causes phase-dependent increases in sympathetic activity

**Figure 2:**
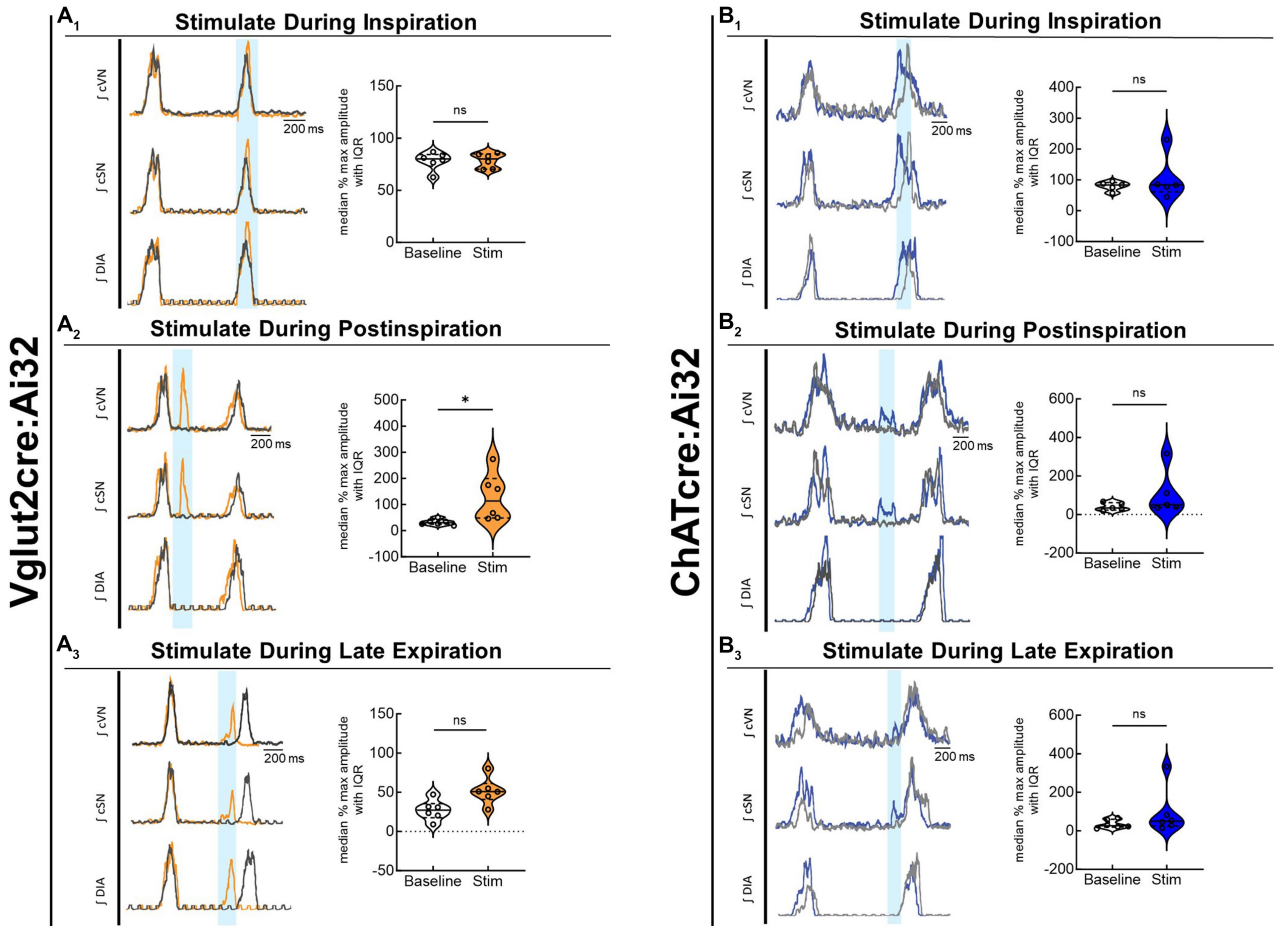
Stimulation of glutamatergic, but not cholinergic, neurons at PiCo causes phase-dependent increases in sympathetic activity. Each panel contains representative traces of raw and integrated (∫) diaphragm (DIA), cervical sympathetic nerve (cSN), and vagus nerve (cVN) during baseline (gray) and optogenetic stimulation (colored) from a control Vglut2cre:Ai32 mouse **(A)**, orange; *n* = 6 (2 male, 4 female) or a control ChATcre:Ai32 mouse **(B)**, blue; *n* = 6 (4 male, 2 female) during each respiratory phase. The shaded light blue area in the trace represents the 200 ms LED activation at PiCo during inspiration (1), postinspiration (2), and late expiration (3). Violin plots illustrate median (solid line) and interquartile range (IQR; dashed lines) while each dot represents data from one mouse. The edges correspond to the data distributions. Repeated measures, two-way ANOVA with a Bonferroni *post hoc* test revealed optogenetic stimulation of glutamatergic neurons (orange trace) at PiCo evoked significant increases in sympathetic activity when the stimulation occurred during postinspiration **(A**_**2**_**)**, but not inspiration **(A**_**1**_**)** or late expiration **(A**_**3**_**)**. Optogenetic stimulation of cholinergic neurons (blue trace) at PiCo did not evoke significant increases in sympathetic activity **(B**_**1**_**–B**_**3**_**)**. *p < 0.05.

To compare the contribution of glutamatergic versus cholinergic neurons in the PiCo area, we optogenetically stimulated Vglut2 or ChAT containing neurons in control mice at random times in the respiratory cycle using previously determined landmarks (Anderson et al., 2016). Figure 2 shows representative data during the *in-vivo* LED stimulations (blue vertical bar) with the colored trace compared to the baseline trace (gray). Repeated measures, two-way ANOVA showed sympathetic responses to optogenetic stimulation (*F*(1, 5) = 11.31; *p* = 0.02) of glutamatergic neurons at PiCo were respiratory phase-dependent (*F*(2, 10) = 4.382; *p* = 0.043). Bonferroni *post hoc* test confirmed that stimulating Vglut2cre:Ai32 neurons at PiCo in control mice evoked significant increases in sympathetic activity during postinspiration (*p* = 0.0035; Figure 2A_2_), but not during late expiration (Figure 2A_3_) or inspiration (Figure 2A_1_). This resulted in significantly decreased discharge duration (Table 2) but did not affect heart rate. By contrast, stimulation of cholinergic neurons did not cause a significant increase in sympathetic activity during any phase of the respiratory cycle (Figure 2B_1_–B_3_; Table 3).

### 3.3 CIH increases respiratory rate and sympathetic discharge in mice

**Figure 3:**
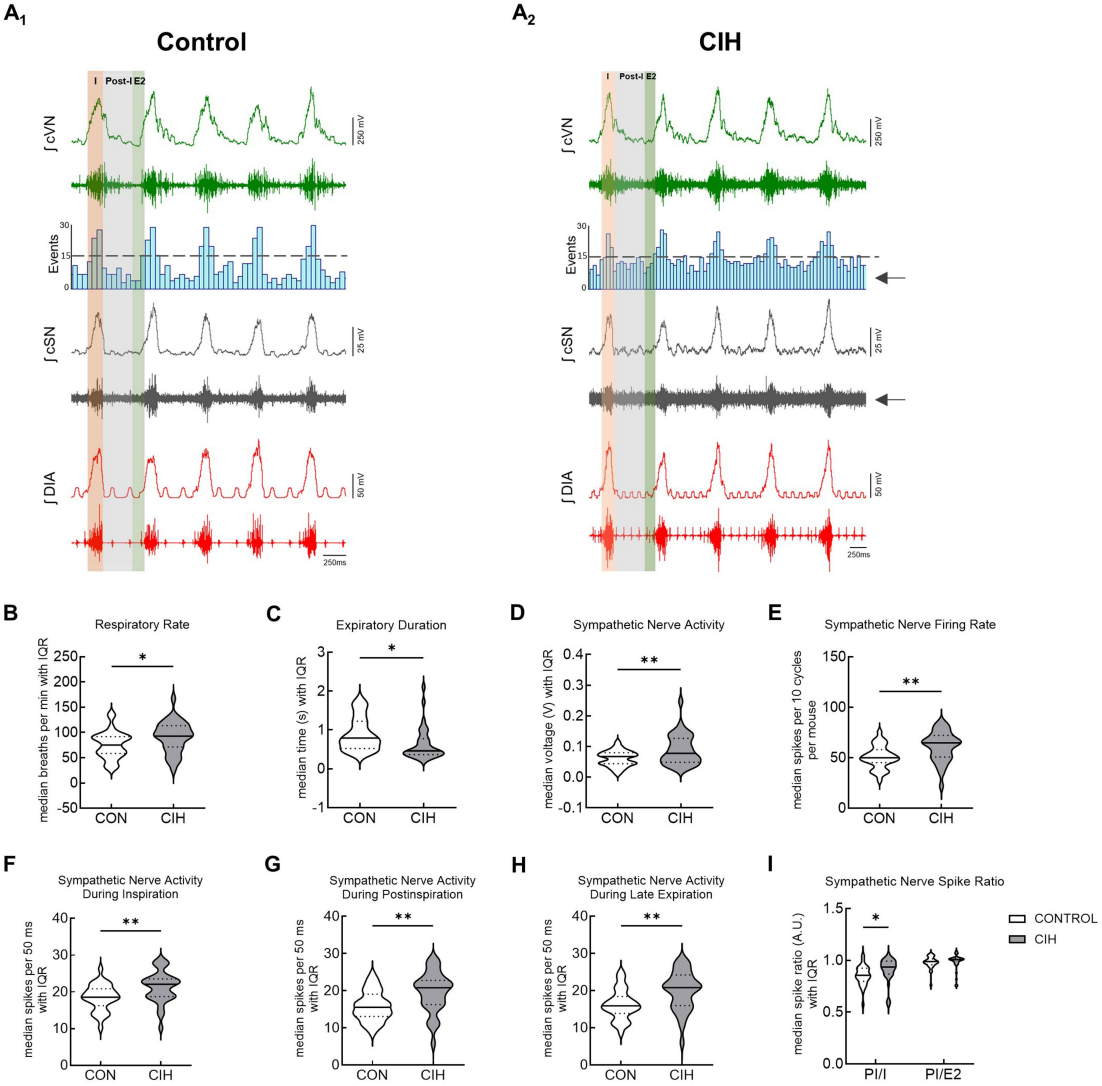
CIH increases respiratory rate and sympathetic discharge in mice. Representative traces of raw and integrated (∫) diaphragm (DIA), cervical sympathetic nerve (cSN), and vagus nerve (cVN) from a urethane anesthetized mouse after control (CON, *n* = 32, **A**_**1**_) or chronic intermittent hypoxia (CIH, *n* = 34, **A**_**2**_) protocols. The phases of the respiratory cycle were determined via the DIA and cVN activity. The orange shaded area in the recording represents inspiration (I). The gray shaded area represents postinspiration (PI). The green shaded area represents the late expiratory phase of the respiratory cycle (E2). The spike distribution is plotted in the histogram and the arrows are pointing to the increased sympathetic nerve activity after CIH. Violin plots show median (solid line) and interquartile range (IQR; dashed lines) while the edges follow the distribution of the data collected from mice that received normoxia or CIH. Unpaired, two-tailed t-tests revealed CIH significantly increased respiratory rate **(B)** and decreased expiratory duration **(C)**, while increasing overall cSN voltage **(D)** and firing rate **(E)**. CIH significantly increased number of cSN spikes during inspiration **(F)**, postinspiration **(G)**, and late expiratory phase **(H)**. Repeated measures, two-way ANOVA revealed the ratio of cSN spikes during postinspiration and inspiration was significantly different while the ratio of spikes during postinspiration and late expiratory phase was not **(I)**. *p < 0.05; **p < 0.01 different from the control group. CON (*n* = 32, 16 male, 16 female); CIH (*n* = 34, 18 male, 16 female).

This study utilized CIH to model the cardiopulmonary effects of obstructive sleep apnea in mice. Figure 3 shows representative traces and summary data from mice that underwent control or CIH conditions. Figures 3A_1,2_ have the respiratory cycle delineated into inspiration (I, orange), postinspiration (Post-I, gray), and late expiration (E2, green) to illustrate how the data were analyzed. The arrows in the CIH representative trace are pointing to the increased sympathetic nerve activity. Both control and CIH mice presented eupneic-like respiratory-sympathetic patterns as shown in representative traces in Figures 3A_1,2_. Mice that underwent the CIH protocol exhibited higher respiratory rate (RR; *p* = 0.025; Figure 3B) and a reduced expiratory duration (*p* = 0.0130; Figure 3C). Furthermore, Figure 3D (*p* = 0.004) and 3E (*p* = 0.001) illustrates that cSN activity was significantly increased across the respiratory cycle following CIH. Compared to control mice, CIH mice exhibited significantly elevated sympathetic activity during inspiration (*p* = 0.0037; Figure 3F), postinspiration (*p* = 0.0019; Figure 3G), and late expiration (*p* = 0.0020; Figure 3H). This was confirmed by the increased postinspiration to inspiration cSN spike ratio (*F*(1, 64) = 83.09; *p* < 0.05) via repeated measures two-way ANOVA and a Bonferroni *post hoc* test (*p* = 0.039; Figure 3I).

### 3.4 Optogenetic stimulation of glutamatergic neurons at PiCo causes increases in sympathetic activity following CIH exposure

**Figure 4:**
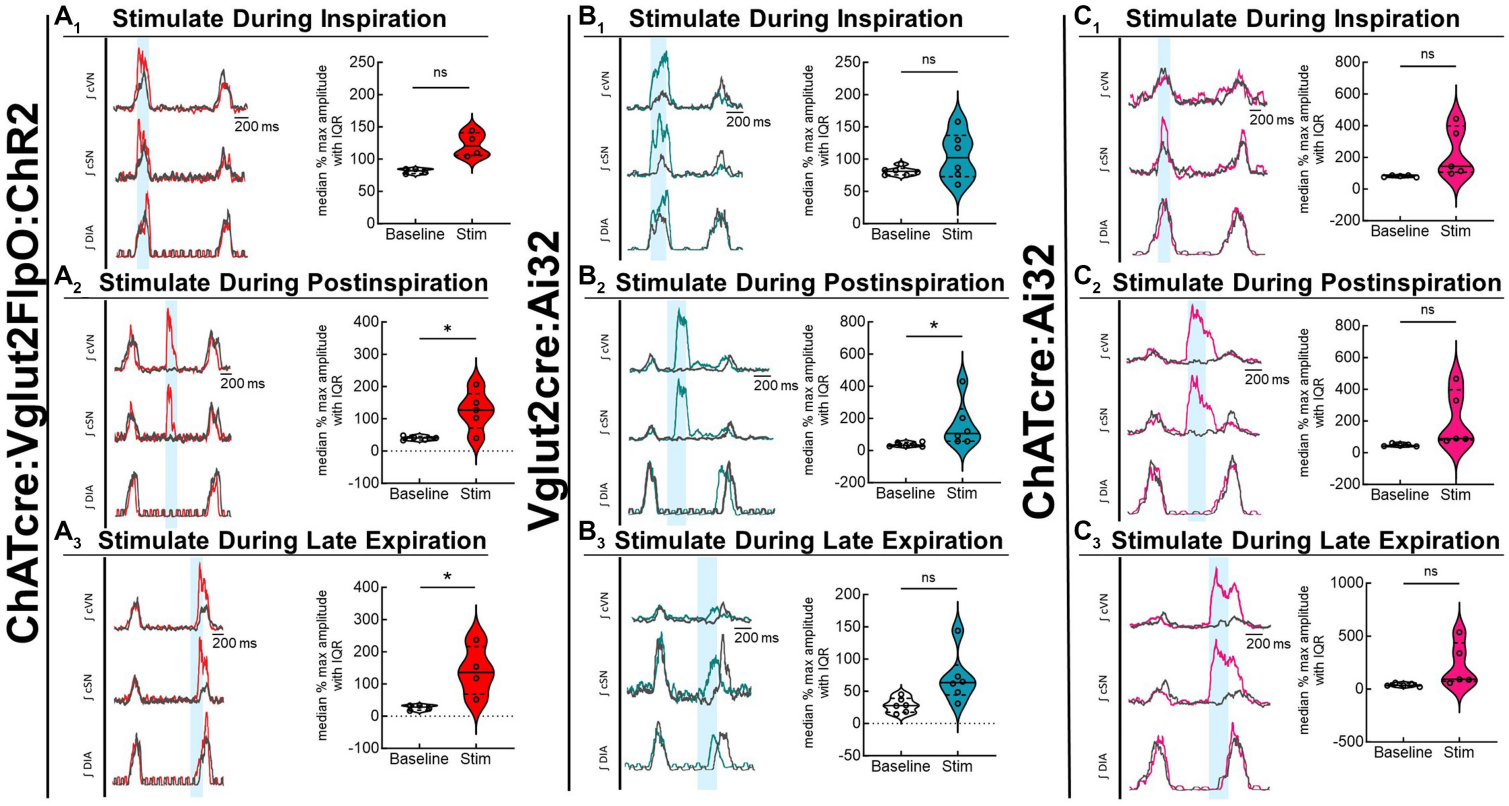
Stimulation of glutamatergic neurons at PiCo causes increases in sympathetic activity after CIH exposure. For each panel, traces of raw and integrated (∫) diaphragm (DIA), cervical sympathetic nerve (cSN), and vagus nerve (cVN) are shown for one ChATcre:Vglut2FlpO:ChR2 **(A)**; *n* = 5 (2 male, 3 female), Vglut2cre:Ai32 **(B)**; *n* = 6 (1 male, 5 female), or ChATcre:Ai32 **(C)**; *n* = 5 (5 male) mouse that underwent the CIH protocol. The 200 ms LED triggered during inspiration (1), postinspiration (2), and late expiration (3) is illustrated by the vertical shaded blue area, with the colored traces representing the response to the optogenetic stimulation relative to the gray baseline trace. Group data are displayed via violin plots with median (solid line), interquartile range (IQR; dashed lines), and data distributions (violin edges). Each dot represents data from one mouse. Mixed effects analysis and Bonferroni *post hoc* test showed optogenetic stimulation of neurons that co-express ChAT and Vglut2 at PiCo (red trace) evoked significant increases in sympathetic activity when the stimulation occurred during postinspiration **(A**_**2**_**)** and late expiration **(A**_**3**_**)**, but not when it occurred during inspiration **(A**_**1**_**)**. Optogenetic stimulation of glutamatergic neurons of Vglut2cre:Ai32 mice at PiCo (blue trace) evoked significant increases in sympathetic activity only during postinspiration **(B**_**2**_**)** as shown via repeated measures, two-way ANOVA and Bonferroni *post hoc* test. No significant differences in sympathetic activity were observed following optogenetic stimulations in the ChATcre:Ai32 mice (pink trace) exposed to CIH **(C**_**1**_**–C**_**3**_**)**. *p < 0.05.

Optogenetic stimulation of excitatory neurons at PiCo was repeated in mice that were exposed to CIH to investigate how CIH affects the role of PiCo in respiratory-sympathetic coupling. Figure 4 illustrates the diaphragm and sympathetic responses to LED activation of excitatory neurons at PiCo area in CIH mice. Following CIH exposure, mixed effects analysis and Bonferroni *post hoc* tests revealed optogenetic stimulation of neurons co-expressing ChAT and Vglut2 increased (*F*(1, 4) = 14.41; *p* = 0.019) the peak cSN amplitude when the stimulus occurred during postinspiration (*p* = 0.021; 4A_2_) and late expiration (*p* = 0.006; 4A_3_), but not inspiration (*p* = 0.318; 4A_1_). The area under the curve and peak amplitudes of the cSN were also significantly increased in response to the LED activation during late expiration (Table 1). Heart rate remained unchanged (Table 1). Repeated measures, two-way ANOVA showed optogenetic stimulation of glutamatergic PiCo neurons evoked significant (*F*(1, 5) = 7.911; *p* = 0.037) increases in cSN activity only during postinspiration (*p* = 0.019; Figure 4B_2_) without affecting heart rate (Table 2). Similar to the control mice (Figure 3B), stimulation of cholinergic PiCo neurons in CIH mice did not significantly increase cSN activity during any phase of the respiratory cycle (Figure 4C_1_–C_3_; Table 3). The maximum amplitudes of cSN responses to LED stimulation of cholinergic, glutamatergic, and cholinergic-glutamatergic neurons were not significantly different between the control and CIH groups.

### 3.5 Optogenetic stimulation of Dbx1 neurons at preBötC evokes inspiration and sympathetic nerve activity

**Figure 5:**
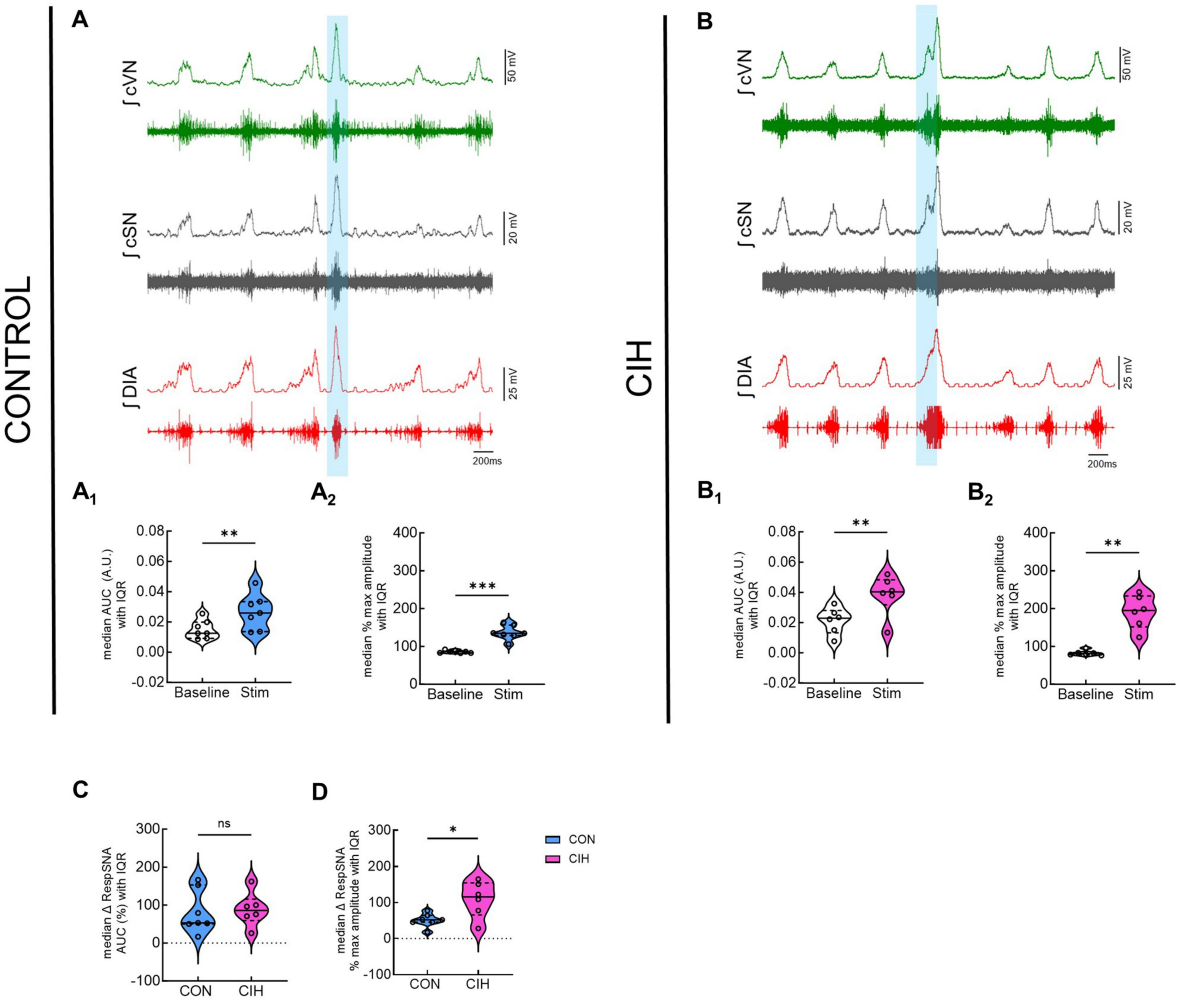
Optogenetic stimulation of Dbx1 neurons at preBötC evokes inspiration and sympathetic nerve activity. Representative data traces of raw and integrated (∫) diaphragm (DIA), cervical sympathetic (cSN), and vagus (cVN) nerve recordings from *in-vivo* preparations of a Dbx1 mouse maintained under normoxia CON, **(A)**; *n* = 7 (6 male, 1 female) and exposed to chronic intermittent hypoxia CIH, **(B)**; *n* = 6 (1 male, 5 female). The shaded blue area represents the 200 ms optogenetic stimulation. Violin plots illustrate median (solid line) and interquartile range (IQR; dashed lines) while each dot represents data from one mouse. Distribution of the data is shown by the plot edges. Optogenetic stimulation evoked significant increases in sympathetic activity for control **(A**_**1**_**,A**_**2**_**)** and CIH mice **(B**_**1**_**,B**_**2**_**)** as confirmed by paired, two-tailed t-tests. The change in sympathetic activity caused by optogenetic stimulation was not significantly different between control and CIH mice **(C)**, however, the percent max amplitude was higher in the CIH group compared to controls **(D)**. *p < 0.05; **p < 0.01; ***p < 0.0001 different from the control group.

Since optogenetic stimulation of excitatory preBötC neurons always evoked an inspiration, it was impossible to stimulate randomly across the respiratory cycle as was done during the PiCo stimulations. Representative traces show that LED activation (blue vertical bar) of Dbx1cre:Ai32 neurons at preBötC increased sympathetic activity in both control mice (Figure 5A) and mice exposed to CIH (Figure 5B). Optogenetic stimulations of 200 ms increased the area under the curve of the integrated cSN activity (*p* = 0.0088; Figure 5A_1_) in control mice. It also increased the maximum amplitude (*p* = 0.0004; Figure 5A_2_) and absolute peak amplitude (*p* = 0.0336; Table 4) without changing cSN discharge duration (Table 4). In mice exposed to CIH, stimulation by LED of Dbx1 neurons significantly increased sympathetic activity (*p* = 0.0044; Figure 5B_1_), maximum amplitude (*p* = 0.0031; Figure 5B_2_), and absolute peak amplitude (*p* = 0.0009; Table 4). No change in cSN discharge duration was observed (Table 4). The overall median cSN responses to optogenetic stimulation were not different between the control and CIH groups (Figure 5C), however, the maximum peak amplitude of cSN following Dbx1 neuron activation at preBötC was increased in mice that were exposed to CIH (*p* = 0.0153; Figure 5D). We observed similar baseline levels in heart rate between control and CIH groups, and optogenetic stimulation did not affect heart rate (Table 4).

### 3.6 Optogenetic stimulation of glutamatergic neurons at preBötC evokes inspiration and sympathetic nerve activity

**Figure 6:**
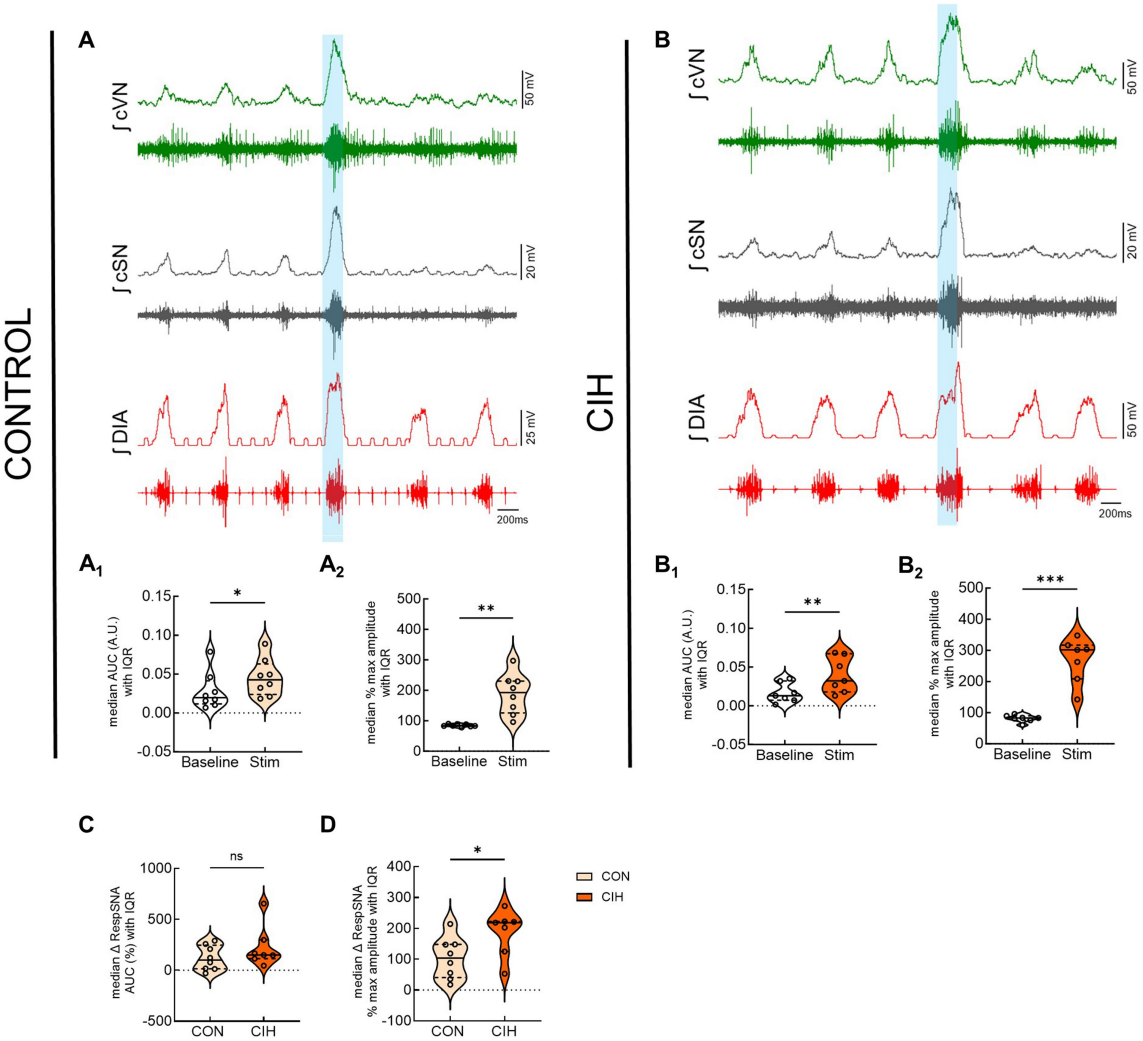
Optogenetic activation of glutamatergic neurons at preBötC evokes inspiration and sympathetic nerve activity. Representative raw and integrated (∫) diaphragm (DIA), cervical sympathetic (cSN), and vagus (cVN) nerve traces recorded from a control CON, **(A)**; n = 8 (5 male, 3 female) and chronic intermittent hypoxia (CIH) Vglut2cre:Ai32 mouse **(B)**; n = 7 (3 male, 4 female). Optogenetic stimulations of 200 ms are shown via the blue shaded region. Violin plots indicate median (solid line) and interquartile range (IQR; dashed lines) and individual mouse data are indicated by the dots. Distribution of the data is shown by the edges of the plots. Paired, two-tailed t-tests show that optogenetic stimulation caused significant increases in sympathetic activity for control **(A**_**1**_**,A**_**2**_**)** and CIH mice **(B**_**1**_**,B**_**2**_**)**. The optogenetic evoked changes in sympathetic activity were not significantly different between control and CIH mice **(C)**, however, the percent max amplitude of the responses was higher in the CIH group compared to the control group **(D)**. *p < 0.05; **p < 0.01; ***p < 0.001 different from the control group.

Figure 6 displays responses to optogenetic stimulation (vertical blue bar) of Vglut2cre:Ai32 neurons at preBötC in control mice (Figure 6A) and CIH mice (Figure 6B). In control mice, LED stimulation significantly increased the cSN area under the curve (*p* = 0.0359; Figure 6A_1_), maximum amplitude (*p* = 0.0032; Figure 6A_2_), and absolute peak amplitude (*p* = 0.0190; Table 2) without altering cSN discharge duration (Table 5). After CIH exposure, stimulation of glutamatergic neurons also significantly increased sympathetic activity (*p* = 0.0064; Figure 6B_1_), cSN maximum amplitude (*p* = 0.0005; Figure 6B_2_) and absolute peak amplitude (*p* = 0.0399; Table 5). Duration of cSN discharge and heart rate were both unaffected (Table 5). Similar to Dbx1 stimulation, the maximal peak amplitude responses to Vglut2 neuronal stimulation were significantly higher in CIH mice compared to control mice (*p* = 0.0357; Figure 6D), although the magnitude of the responses were not (Figure 6C).

### 3.7 Optogenetic stimulation of inhibitory neurons at preBötC decreases respiratory rate but does not alter sympathetic activity

To test for preBötC inhibitory control over sympathetic output, this study optogenetically stimulated Vgatcre:Ai32 neurons for 10 s at preBötC area. Figure S1 shows representative data from control (Supplementary Figure S1A) and CIH (Supplementary Figure S1C) mice. LED activation of inhibitory neurons decreased respiratory rate without altering the sympathetic discharge in both control (Supplementary Figure S1B) and CIH groups (Supplementary Figure S1D).

## 4 Discussion

Exposure to CIH altered the respiratory modulation of sympathetic discharge in urethane anesthetized, freely breathing mice. Mice exposed to CIH exhibited abnormal breathing patterns, disrupted respiratory-sympathetic coupling, and significantly elevated sympathetic activity. Here we provide new evidence that PiCo contributes to respiratory-sympathetic coupling in control mice and mice exposed to CIH. Optogenetic activation of glutamatergic-cholinergic neurons in PiCo region evoked increases in sympathetic activity throughout the respiratory cycle. For CIH mice, sympathetic activity was increased in response to LED stimulation of glutamatergic-cholinergic neurons during postinspiration and late expiration phases. Thus, optogenetic activation of PiCo specific neurons increased sympathetic activity in both control and CIH mice. To differentiate the effects of stimulating glutamatergic versus cholinergic neurons within PiCo, we found that stimulating glutamatergic neurons in PiCo evoked increases in sympathetic activity only during the postinspiratory phase in control mice. Following CIH, optogenetic stimulation of glutamatergic PiCo neurons also only evoked significant increases in sympathetic activity during the postinspiratory phase. Interestingly, stimulating only cholinergic PiCo neurons did not evoke an elevated sympathetic response compared to baseline in control or CIH mice. Stimulating Dbx1 or glutamatergic neurons in preBötC of control and CIH mice evoked inspiratory activity concomitant with increased sympathetic activity. Overall, sympathetic activity was differentially increased by phasic inputs from optogenetically stimulated excitatory respiratory neurons in the ventrolateral medulla. These findings, along with limitations of this study, will be discussed.

### 4.1 Chronic intermittent hypoxia alters respiratory-sympathetic coupling

CIH models the frequent hypoxic episodes that occur in humans with obstructive sleep apnea (Barnes et al., 2022) and other disorders of dysautonomia (Ramirez et al., 2023). In the present study, mice that underwent the CIH protocol exhibited higher baseline respiratory rate with reduced expiratory duration, as well as increased cervical sympathetic nerve activity (Figure 1). Furthermore, this sympathetic overactivity occurred throughout the respiratory cycle, indicating a decoupling of normal respiratory-sympathetic modulation (Figure 1). These results are consistent with the CIH-induced cardiopulmonary changes observed in rats (Zoccal et al., 2008; Molkov et al., 2011; Moraes et al., 2012; Karlen-Amarante et al., 2023) and increases in blood pressure seen in mice (Campen et al., 2005). These findings support the interpretation that CIH secondary to obstructive sleep apnea is a risk factor for developing high blood pressure due to increased sympathetic drive.

The mechanisms underlying the disruption of normal respiratory-sympathetic coupling following CIH remain unclear. Early life exposure to CIH elicits higher levels of blood pressure and a greater carotid body sensory response during subsequent exposure to hypoxia (Nanduri et al., 2012). This has been attributed to dysregulation of hypoxia-inducible factor (HIF) 1α and 2α which are oxygen sensitive transcriptional factors that regulate the homeostatic responses to hypoxia (Prabhakar and Semenza, 2012). Consistent with the results of the present study, presympathetic neurons in the RVLM of rats exposed to CIH show increased resting activity and firing variability (Karlen-Amarante et al., 2023). This increased firing activity of RVLM presympathetic neurons is not due to changes in the intrinsic electrophysiological properties induced by CIH (Almado et al., 2014). Rather, it is due to a plethora of factors that collectively alter presympathetic neuron activity and disrupt respiratory-sympathetic coupling. These factors include overexpression of HIF-1α (Karlen-Amarante et al., 2023), sensitization of central (Molkov et al., 2011) and peripheral chemoreceptors (Prabhakar et al., 2005, 2023), and dysregulation of astrocyte homeostatic control of blood pressure (Marina et al., 2020). However, more studies are needed to elucidate the complex mechanisms underlying sympathetic overactivation caused by CIH (Guyenet et al., 2020; Barnes et al., 2022).

### 4.2 Optogenetic activation of excitatory neurons in respiratory networks *in-vivo* increases sympathetic activity

In addition to sympathetic overactivity, this study also demonstrates that phasic optogenetic stimulation of excitatory neurons in PiCo and preBötC increased sympathetic activity. Mice that received the CIH protocol showed larger inspiratory related peak sympathetic bursts during optogenetic stimulation of Dbx1 (Figure 5D) and Vglut2 (Figure 6D) neurons at preBötC. This suggests that CIH caused an increase in excitatory tone to the RVLM presympathetic neurons from preBötC which altered respiratory-sympathetic modulation. The increased sympathetic activity following stimulation of excitatory neurons in preBötC complements existing evidence that neurons in the preBötC send direct projections to presympathetic C1 neurons (Moraes et al., 2013; Dempsey et al., 2017; Menuet et al., 2017, 2020) where they provide excitatory and inhibitory drive to vasomotor sympathetic activity. This tonic drive likely occurs via presympathetic C1 neurons whose activity positively correlates with inspiration (Marina et al., 2011; Moraes et al., 2013; Menuet et al., 2017). In rats, prolonged optogenetic stimulation of inhibitory preBötC neurons decreases sympathetic activity (Menuet et al., 2020) via suppression of the phasic activity of inhibitory expiratory preBötC neurons (Carroll et al., 2013; Baertsch et al., 2018), indicating the preBötC is critically involved in tonic regulation of the presympathetic neuronal discharge through a combination of excitatory and inhibitory neuronal mechanisms. However, in the present study, stimulation of GABAergic neurons in preBötC of mice did not yield any significant decreases in sympathetic activity compared to baseline (Supplementary Figure S1). This contrasting result may be attributed to differences between species, stimulation durations, or inhibitory neuronal genetic markers targeted.

In addition to preBötC, PiCo may also excite presympathetic neurons via its projections to the RVLM (Dempsey et al., 2017). Stimulating glutamatergic or cholinergic-glutamatergic neurons in the PiCo area had a more pronounced effect on sympathetic activity than stimulating excitatory neurons in preBötC. For example, excitatory preBötC stimulations caused a spike in sympathetic activity and a concurrent inspiration (Figures 5, 6), whereas only stimulating glutamatergic or more specifically, the cholinergic-glutamatergic PiCo neurons caused a disassociation of normal respiratory-sympathetic coupling by evoking a spike in sympathetic activity independent of inspiration (Figures 2–4). Moreover, optogenetic stimulation of cholinergic-glutamatergic PiCo neurons significantly increased sympathetic activity at all phases of the respiratory cycle and not only during the postinspiratory phase. However, since PiCo is typically only active in phase with postinspiration (Anderson et al., 2016), this region likely contributes to the postinspiratory component of respiratory-sympathetic coupling under control conditions. We recently demonstrated via respiratory phase reset analysis that activation of glutamatergic-cholinergic PiCo neurons during inspiration or at the beginning of postinspiration is highly likely to reset the respiratory rhythm and trigger a swallow, suggesting that PiCo plays a role in swallow-breathing coordination (See Huff et al., 2023; Figure 3A). In the present study, similar swallow responses and corresponding cSN activity were observed during optogenetic stimulation of PiCo, however, these responses were not included in the analysis, allowing the characterization of PiCo contribution to cardiorespiratory coupling separately from swallow behavior.

Following CIH, previous studies reported distinct firing patterns of RVLM neuronal activity during different phases of the respiratory cycle (Moraes et al., 2013; Zoccal et al., 2019) and overall increases in RVLM activity throughout the respiratory cycle (Zoccal et al., 2008; Moraes et al., 2013; Zoccal et al., 2019), leading to high blood pressure (Zoccal et al., 2007a,b; Souza et al., 2016; Bazilio et al., 2019). Since optogenetic stimulation of excitatory neurons in both preBötC and PiCo increased sympathetic activity, the activity from these two rhythmogenic networks may differentially modulate sympathetic activity during inspiration and postinspiration, respectively. PiCo is not always active throughout the respiratory cycle (Anderson et al., 2016), and postinspiratory motor activity is often missing in phrenic nerve recording of rodents (Dutschmann and Paton, 2002), which may explain why sympathetic activity is dominated by diaphragm burst activity under baseline conditions. The present data show that PiCo and preBötC both evoke sympathetic burst activity, but further studies are necessary to unravel their underlying connectivity to the RVLM sympathetic neurons. RVLM sympathetic premotor neurons include both C1 and non-C1 neurons, which differ in their functional and neurochemical phenotypes and could play differing roles in the generation of sympathetic nerve activity (Schreihofer and Guyenet, 1997; Stornetta et al., 2001).

### 4.3 Limitations and conclusions

There are several limitations to the present study that merit consideration. First, there are differences in healthy sympathetic patterns between rodents and humans. Specifically, sympathetic activity dominates during expiration instead of inspiration in humans (Zoccal et al., 2008, 2009; Karlen-Amarante et al., 2023). It is conceivable that, in humans, inspiratory driven sympathetic activity is largely inhibited by vagal afferents, as suggested by experimentally induced lung inflations which inhibit pre-and postganglionic sympathetic nerve activity in anesthetized or decerebrate, vagally intact laboratory animals (Seals et al., 1990). During quiet resting breathing, muscle sympathetic nerve activity (MSNA) is highest at end-expiration and lowest at end-inspiration (Hagbarth and Vallbo, 1968; Katayama et al., 2021). However, the respiratory modulation of MSNA is independent of changes in blood pressure and baroreceptor activity (Seals et al., 1990; Macefield and Wallin, 1995).

Second, measures of respiratory and sympathetic activity contained a high amount of variability which may have increased the likelihood of committing a type-1 error. Due to the nature of the methods by which the data must be collected, a high amount of variability is expected, as mentioned in the “Optogenetic stimulation and data analysis” section. However, every effort was made to quantify differences in dependent measures without unnecessary data transformations. No outliers were removed, and any missing data was missing at random. Despite this variability, significant effects due to optogenetic stimulations or CIH were observed, underscoring the robust nature of the phenomena.

Third, due to technical limitations associated with the exposed ventral brainstem *in-vivo* preparation, blood pressure was not included as a dependent measure in this study. Despite the absence of blood pressure data, we did not see any changes in the HR associated with the optogenetic stimulations. This is likely because the latency of the cSN response to the optogenetic stimulations was too short to be triggered by periphery circuits, suggesting a central origin of the sympathetic response. Previous studies have correlated sympathetic overactivity with the incidence of hypertension following CIH exposure in rodents (Greenberg et al., 1985; Fletcher, 2001; Zoccal et al., 2007b; Prabhakar and Kumar, 2010; Moraes et al., 2012) and humans (Somers et al., 1995), and these findings contribute to the interpretation of the present data. Most studies of sympathetic activity are performed using the thoracic, renal, or splanchnic sympathetic branches which all present a phasic activity modulation that increases during the end of inspiration and decreases throughout the respiratory cycle. Although the cSN activity in the present study has a monophasic discharge, this difference is likely due to the differential channeling of A- and C-fiber inputs to distinct populations of vasomotor neurons (McMullan et al., 2008).

Forth, it is a well-known limitation of the optogenetic technique that collateral activation of neurons outside of the target area may occur due to light scatter through tissue. It is possible other nearby glutamatergic neurons are stimulated when targeting the PiCo or preBötC areas, since glutamatergic neurons are distributed throughout the entire VRC (Bush and Ramirez, 2024). It is also an issue when stimulating only cholinergic PiCo neurons. Adjacent cholinergic neurons located in the Nucleus ambiguus will also be stimulated during stimulation of the cholinergic-glutamatergic interneurons in the PiCo region. Precautions were taken to circumvent this possibility such as titrating the LED power and using established, previously published landmarks for the position of the LED optrodes (Anderson et al., 2016; Huff et al., 2023). Furthermore, stimulations of cholinergic-glutamatergic neurons were performed to specifically limit stimulation only to PiCo neurons, as only PiCo neurons have been shown to express both ChAT and Vglut2 excitatory markers in that brainstem region (Anderson et al., 2016; Huff et al., 2023). PiCo is a relatively new region of interest, and future studies are necessary to fully elucidate the exact function, nomenclature, anatomical connections, and contribution of these neurons to respiratory rhythm, swallowing, and laryngeal behaviors as well other autonomic functions.

This study demonstrates that PiCo and preBötC contribute to respiratory-sympathetic coupling and that exposure to CIH alters this coupling. CIH exposure disrupted breathing, increased baseline sympathetic tone, and altered respiratory phase-dependent modulation of sympathetic activity. Optogenetically stimulating preBötC and PiCo glutamatergic neurons and PiCo specific cholinergic-glutamatergic neurons significantly increased sympathetic activity. Taken together, the present study suggests that the heightened sympathetic activity to the cardiovascular system in mice exposed to CIH may be driven by increased excitatory tone from the respiratory networks. Specifically, the current results support the interpretation that CIH causes a cascade of events which begin with an overactive carotid body (Nanduri et al., 2012) and lead to RLVM hyperactivity that is exacerbated by an increased excitatory drive from PiCo and preBötC. However, it is unlikely that these respiratory complexes are the only contributing factors to the RVLM overactivity, given that CIH is known to cause a variety of other changes in the respiratory network (Zoccal et al., 2008; Molkov et al., 2011; Moraes et al., 2012, 2013; Bazilio et al., 2019; Zoccal et al., 2019; Bittencourt-Silva et al., 2020; Karlen-Amarante et al., 2023). These findings are consistent with the pathogenesis of obstructive sleep apnea related hypertension, which is a risk factor for developing life-threatening cardiovascular diseases.

## Data availability statement

The raw data supporting the conclusions of this article will be made available by the authors, without undue reservation.

## Ethics statement

The animal study was approved by Seattle Childrens Research Institute Institutional Animal Care and Use Committee. The study was conducted in accordance with the local legislation and institutional requirements.

## Author contributions

MK-A: Conceptualization, Data curation, Formal analysis, Investigation, Methodology, Software, Validation, Writing – original draft, Writing – review & editing. ZG: Conceptualization, Data curation, Formal analysis, Methodology, Software, Validation, Visualization, Writing – original draft, Writing – review & editing. AH: Conceptualization, Data curation, Investigation, Methodology, Software, Writing – original draft, Writing – review & editing. LO: Conceptualization, Data curation, Formal analysis, Investigation, Methodology, Software, Writing – original draft, Writing – review & editing. J-MR: Conceptualization, Data curation, Formal analysis, Funding acquisition, Investigation, Methodology, Project administration, Resources, Software, Supervision, Validation, Visualization, Writing – original draft, Writing – review & editing.
