# Supplementary material for: Postinspiratory and preBötzinger complexes contribute to respiratory-sympathetic coupling in mice before and after chronic intermittent hypoxia
Source: Front Neurosci. 2024 May 6;18:1386737. doi: 10.3389/fnins.2024.1386737 (PMC11107097; doi:10.3389/fnins.2024.1386737)
Supplement: Supplementary file 1 [file Table_1.DOCX]

Supplemental Table 1. Results from repeated-measures, two-way analysis of variance reported in the figures. Each table corresponds to a specific figure.

| **4.1 Stimulating cholinergic-glutamatergic neurons at PiCo increases sympathetic activity** | | | | |
| --- | --- | --- | --- | --- |
| 1. *ChATcre:Vglut2FlpO:ChR2- Control, Repeated Measures Two-Way ANOVA* | | | | |
| Source | Sum of squares | Mean square | F (DFn, DFd) | P value |
| Optogenetic (Opto) stimulation (stim) | 55524 | 55524 | F (1, 4) = 9.981 | P=0.0342* |
| Respiratory phase | 7572 | 3786 | F (2, 8) = 34.06 | P=0.0001* |
| Opto stim x Respiratory phase | 1078 | 539.0 | F (2, 8) = 5.730 | P=0.0286* |
| Subject x Opto stim | 22252 | 5563 |  |  |
| Subject x Respiratory phase | 889.3 | 111.2 |  |  |
| Subject | 19926 | 4982 |  |  |
| Residual | 752.5 | 94.07 |  |  |

| **4.2 Optogenetic stimulation of glutamatergic, but not cholinergic, neurons at PiCo causes phase-dependent increases in sympathetic activity** | | | | |
| --- | --- | --- | --- | --- |
| 1. *Vglut2cre:Ai32- Control, Repeated Measures Two-Way ANOVA* | | | | |
| Source | Sum of squares | Mean square | F (DFn, DFd) | P value |
| Optogenetic (Opto) stimulation (stim) | 14601 | 14601 | F (1, 5) = 11.31 | P=0.0200* |
| Respiratory phase | 12866 | 6433 | F (2, 10) = 4.382 | P=0.0430* |
| Opto stim x Respiratory phase | 14919 | 7459 | F (2, 10) = 5.425 | P=0.0254* |
| Subject x Opto stim | 6454 | 1291 |  |  |
| Subject x Respiratory phase | 14680 | 1468 |  |  |
| Subject | 9653 | 1931 |  |  |
| Residual | 13750 | 1375 |  |  |
| 1. *ChATcre:Ai32- Control, Mixed Effects Analysis* | | | | |
| Source | SD | Variance | F (DFn, DFd) | P value |
| Opto stim |  |  | F (1, 5) = 1.764 | P=0.2415 |
| Respiratory phase |  |  | F (2, 10) = 1.363 | P=0.2997 |
| Opto stim x Respiratory phase |  |  | F (2, 6) = 3.909 | P=0.0819 |
| Subject | 39.05 | 1525 |  |  |
| Subject x Opto stim | 58.75 | 3451 |  |  |
| Subject x Respiratory phase | 10.95 | 119.8 |  |  |
| Residual | 21.21 | 449.7 |  |  |

| **4.3 CIH increases respiratory rate and sympathetic discharge in mice** | | | | |
| --- | --- | --- | --- | --- |
| 1. *Sympathetic Nerve Spike Ratio- Repeated Measures Two-Way ANOVA* | | | | |
| Source | Sum of squares | Mean square | F (DFn, DFd) | P value |
| Phase Ratio x Treatment | 0.01759 | 0.01759 | F (1, 64) = 4.220 | P=0.0440* |
| Phase Ratio | 0.3463 | 0.3463 | F (1, 64) = 83.09 | P<0.0001* |
| Treatment | 0.02807 | 0.02807 | F (1, 64) = 2.346 | P=0.1305 |
| Subject | 0.7659 | 0.01197 | F (64, 64) = 2.872 | P<0.0001* |
| Residual | 0.2667 | 0.004167 |  |  |

| **4.4 Optogenetic stimulation of glutamatergic neurons at PiCo causes increases in sympathetic activity following CIH exposure** | | | | |
| --- | --- | --- | --- | --- |
| 1. *ChATcre:Vglut2FlpO:ChR2- CIH, Mixed Effects Analysis* | | | | |
| Source | SD | Variance | F (DFn, DFd) | P value |
| Opto stim |  |  | F (1, 4) = 14.41 | P=0.0192* |
| Respiratory phase |  |  | F (2, 8) = 1.727 | P=0.2380 |
| Opto stim x Respiratory phase |  |  | F (2, 4) = 4.439 | P=0.0965 |
| Subject | 8.701 | 75.70 |  |  |
| Subject x Opto stim | 31.05 | 964.2 |  |  |
| Subject x Respiratory phase | 7.956 | 63.31 |  |  |
| Residual | 21.49 | 461.8 |  |  |
| 1. *Vglut2cre:Ai32- CIH, Repeated Measures Two-Way ANOVA* | | | | |
| Source | Sum of squares | Mean square | F (DFn, DFd) | P value |
| Optogenetic (Opto) stimulation (stim) | 35512 | 35512 | F (1, 5) = 7.911 | P=0.0374* |
| Respiratory phase | 17418 | 8709 | F (2, 10) = 2.538 | P=0.1284 |
| Opto stim x Respiratory phase | 16803 | 8402 | F (2, 10) = 2.193 | P=0.1623 |
| Subject x Opto stim | 22444 | 4489 |  |  |
| Subject x Respiratory phase | 34311 | 3431 |  |  |
| Subject | 23282 | 4656 |  |  |
| Residual | 38304 | 3830 |  |  |
| 1. *ChATcre:Ai32- CIH, Repeated Measures Two-Way ANOVA* | | | | |
| Source | Sum of squares | Mean square | F (DFn, DFd) | P value |
| Optogenetic (Opto) stimulation (stim) | 203228 | 203228 | F (1, 4) = 4.484 | P=0.1016 |
| Respiratory phase | 4529 | 2265 | F (2, 8) = 4.202 | P=0.0566 |
| Opto stim x Respiratory phase | 1537 | 768.5 | F (2, 8) = 2.016 | P=0.1954 |
| Subject x Opto stim | 181306 | 45326 |  |  |
| Subject x Respiratory phase | 4311 | 538.9 |  |  |
| Subject | 214091 | 53523 |  |  |
| Residual | 3050 | 381.2 |  |  |
