# Supplementary figures and images for: Postinspiratory and preBötzinger complexes contribute to respiratory-sympathetic coupling in mice before and after chronic intermittent hypoxia
Source: Front Neurosci. 2024 May 6;18:1386737. doi: 10.3389/fnins.2024.1386737 (PMC11107097; doi:10.3389/fnins.2024.1386737)

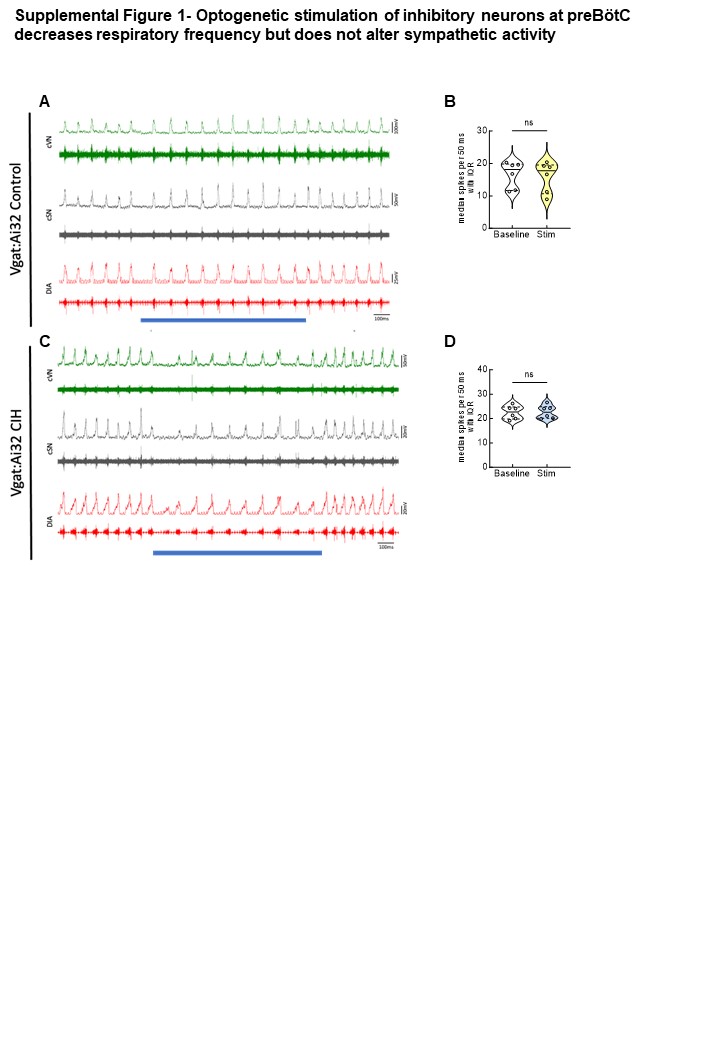

Supplement: Supplementary file 2 [file Image_1.JPEG]
